# Case of Softening of the Spinal Marrow

**Published:** 1838-08-15

**Authors:** 


### CLINICAL REPORTS.

### PENNSYLVANIA HOSPITAL.

*Case of softening of the Spinal Marrow.*

Robert B., aged 17 years, was admitted into the hospital, 12th September, 1837, with complete paralysis of all the limbs, which had come on a short time previously, during his convalescence from, or in the course of, an attack of typhoid fever. He remained in the hospital, in this condition, unable to leave his bed, for about a year, at the end of which period he died, Aug. 2, 1838. Dr. Stewardson saw him at the time of his admission and had charge of him for a month previous to his death, but visited him only once or twice during the intermediate time. From first to last his intellect was perfectly clear; he had no pain in the head; his senses were perfect; his countenance natural; and he had no spasms or convulsions. During a part of the time his bladder and rectum were involved in the paralysis, his stools being involuntary, and he being unable to pass his urine. He never admitted the existence of any pain in his back, and the bones of his spine appeared to be in a natural condition. Although the loss of power was complete or nearly so, the sensation in the limbs was preserved. Some time after his admission, the limbs, especially the upper extremities, became the seat of permanent contractions, the fore-arm being flexed upon the arm, the hand upon the fore-arm, and the fingers upon the hand. For some time previous to his death, the power of the bladder and rectum was restored, and there was a slight return of motion in the limbs. The emaciation, however, progressed, and the patient gradually sank without the occurrence of any other remarkable symptoms, except a slight cough, with, perhaps, a little pain in the chest. These symptoms, however, were so slight, that, during the month Dr. S. had charge of him, (the last of his life,) my attention was not arrested by them, and he did not auscult him.

*Autopsy* fourteen hours after death:—extreme emaciation.

Brain:—Membranes presenting nothing remarkable; several ounces of serum, in the cavity of the arachnoid. Substance of the brain perfectly healthy. Vault and septum lucidum softened, as well as the surface of the thalami and corpora striata, forming the walls of the ventricles. Some effusion into the ventricles. Nothing else worthy of note.

Spinal Marrow:—Some adhesions, laterally, between the opposing surfaces of arachnoid. The pia mater with great difficulty separated from the substance of the spinal marrow, portions of which adhered to it. Upon dividing it along the posterior fissure, the substance of the spinal marrow was found perfectly pulpy, and of a milk white colour. This softening extended throughout the whole length of the column, but was most marked inferiorly, less so in the cervical, and least of all in the dorsal portion. It was limited to the posterior columns, which were softened throughout, its limits being distinctly marked by the posterior horns of the cineritious substance, which was rather paler and less distinct than natural. The anterior columns were of natural consistence and colour.

A considerable quantity of tuberculous matter was infiltrated into both lungs, crude in the right, softened, in part, in the left.

The condition of the heart was natural, except some valvular thickenings. Liver large and fatty. Spleen small and of natural aspect. A superficial examination of the stomach and intestines presented nothing very remarkable. The glands of Peyer were not examined.

